# Screening for HFpEF in pacemaker patients: Study design and protocol of the PM-HFpEF study

**DOI:** 10.1371/journal.pone.0349667

**Published:** 2026-06-12

**Authors:** Elisabeth Santos, Rafael Teixeira, João Almeida, Paulo Fonseca, Marco Oliveira, Helena Gonçalves, João Primo, Sílvia Diaz, António Barros, Eduardo Vilela, Francisco Sampaio, Francisca Saraiva, Mário Oliveira, Ricardo Fontes de Carvalho

**Affiliations:** 1 Department of Cardiology, Local Health Unit of Gaia and Espinho, Vila Nova de Gaia, Portugal; 2 RISE-Health, Department of Surgery and Physiology, Faculty of Medicine, University of Porto, Alameda Prof. Hernâni Monteiro, Porto, Portugal; 3 Local Health Unit of Central Lisbon – São José Hospital, Portugal; 4 Department of Medicine, Faculty of Medicine, University of Porto, Porto, Portugal; Tehran University of Medical Sciences, IRAN, ISLAMIC REPUBLIC OF

## Abstract

**Background:**

Heart failure with preserved ejection fraction (HFpEF) is common in older multimorbid patients and is associated with substantial morbidity and mortality. Pacemaker (PM) patients may be particularly vulnerable given the clustering of conventional HFpEF risk factors and the hemodynamic effects of long-term right ventricular pacing. Nevertheless, HFpEF is rarely systematically assessed in device clinics.

**Objectives:**

To determine the prevalence of HFpEF in a cohort of permanent PM patients using a structured guideline-aligned screening protocol and to characterize their clinical, functional, and pacing-related profiles.

**Methods:**

PM-HFpEF is a single-center, prospective, cross-sectional study conducted at a tertiary hospital in Portugal. Eligible adults with conventional transvenous pacemakers and right ventricular apical pacing implanted between January 2018 and December 2022 will be invited to attend a one-day screening visit. The protocol includes clinical assessment, N-terminal pro-B-type natriuretic peptide testing, 12-lead electrocardiography, comprehensive transthoracic echocardiography, routine device interrogation, frailty assessment (the Portuguese-validated version of the Edmonton Frail Scale), and health-related quality-of-life evaluation using the 12-item Kansas City Cardiomyopathy Questionnaire. Exercise diastolic stress echocardiography using an upper-limb ergometer will be performed when resting findings are inconclusive. HFpEF will be adjudicated according to the 2021 European Society of Cardiology diagnostic definition.

**Discussion:**

The PM-HFpEF study is designed to provide robust estimates of HFpEF prevalence within a real-world PM population, together with detailed characterization of their clinical and device-related profiles. This information may help inform future clinical assessment and research strategies for this often-overlooked yet clinically significant group of patients.

## Introduction

Heart failure with preserved ejection fraction (HFpEF) is defined by signs or symptoms of heart failure (HF), a left ventricular ejection fraction (LVEF) ≥50%, and objective evidence of elevated filling pressures supported by structural or functional cardiac abnormalities and increased natriuretic peptides [1]. HFpEF accounts for approximately half of all HF cases and is associated with impaired quality of life, recurrent hospitalizations, and increased mortality [1]. Its prevalence is rising, mainly driven by population aging and the increasing burden of hypertension, diabetes, obesity, coronary artery disease, and chronic kidney disease [2–4].

Patients with permanent pacemakers may be particularly vulnerable to HFpEF [5–7]. This population is typically older and carries a high burden of comorbidities and cardiovascular risk factors (CVRFs), common to the HFpEF profile [6,8]. In addition, long-term right ventricular (RV) pacing may disrupt electrical and mechanical synchrony, which may increase filling pressures and contribute to adverse ventricular remodeling [9–11]. These pacing-related effects are rarely assessed during routine device follow-up, which may delay optimization of device programming and broader clinical assessment [10,12,13].

Despite the growing awareness of this overlap, evidence in this group remains limited [14,15]. HFpEF diagnostic studies rarely include detailed assessments of pacing-related electrical or mechanical parameters [5,14,15]. Furthermore, pacemaker (PM) patients remain consistently under-represented in major HFpEF trials [16–19]. This persistent gap suggests that the presence of HFpEF may be overlooked in this population and highlights the need for a structured diagnostic approach tailored to the challenges of paced rhythms.

To our knowledge, no systematic HFpEF screening approach based on the 2021 ESC diagnostic definition has been applied specifically to PM patients. The Pacemaker Heart Failure with Preserved Ejection Fraction (PM-HFpEF) study aims to address this gap by estimating the prevalence of HFpEF in a cohort of PM patients using a structured, single-day screening protocol aligned with ESC criteria. The study will also characterize clinical, functional, and pacing-related profiles to enhance diagnostic accuracy and support timely clinical reassessment.

## Materials and methods

This manuscript reports the study protocol of the PM-HFpEF prospective cross-sectional screening study.

### Study design and setting

PM-HFpEF is a single-center, prospective cross-sectional study conducted at a tertiary hospital in Portugal. The study applies a structured, single-visit screening protocol aligned with the 2021 ESC diagnostic definition for HFpEF [1]. The design aims to standardize clinical, imaging, laboratory, and device-based assessments within a unified diagnostic framework.

### Study population

All adults who underwent a permanent PM implantation with conventional transvenous RV apical pacing at the study center between January 2018 and December 2022 (n = 1802) will be screened for eligibility. Inclusion criteria require active clinical follow-up at the device clinic, the absence of exclusion criteria, and written informed consent. Eligible patients will be included, irrespective of pacing indication, device programming mode, or atrial/ventricular pacing burden. Devices from multiple vendors (Medtronic, Boston Scientific, Abbott, MicroPort, and Biotronik) will be included to reflect routine clinical practice. Full exclusion criteria are presented in **Table 1**.

**Table 1:** Clinical exclusion criteria applied in the PM-HFpEF study.

Clinical domain	Exclusion criterion
**Institutionalization**	Residence in institutional care settings (nursing homes, prisons, or military facilities).
**Functional or cognitive limitations**	Severe impairment precluding informed consent or study participation (e.g., advanced dementia, major aphasia, or severe uncorrected sensory deficits).
**Cardiac function**	Documented LVEF <50% before enrolment or at time of PM implantation.
**Coronary artery disease**	Myocardial infarction within the last 6 months.
**Cardiomyopathy**	Known infiltrative cardiomyopathy; hypertrophic cardiomyopathy.
**Valvular heart disease**	Severe valvular disease as defined by most recent echocardiographic criteria (e.g., aortic stenosis, severe regurgitation) [20–22]; presence of mitral valve prostheses or annuloplasty rings.
**Renal function**	Advanced chronic kidney disease: estimated glomerular filtration rate (eGFR) < 15 mL/min/1.73 m².[23]
**Oncological disease**	Active cancer currently receiving systemic treatment.
**Pulmonary disease**	Severe respiratory disease (e.g., COPD or other chronic respiratory conditions associated with resting hypoxemia or requiring long-term oxygen therapy).[24]
**Device therapy**	CRT devices, ICDs, leadless pacemakers, conduction system pacing (His-bundle pacing or left bundle branch area pacing), epicardial leads, and any non-apical right ventricular pacing position.

**Abbreviations:** CKD, chronic kidney disease; CKD-EPI, chronic kidney disease epidemiology collaboration; COPD, chronic obstructive pulmonary disease; CRT, cardiac resynchronization therapy; eGFR, estimated glomerular filtration rate; LVEF, left ventricular ejection fraction; PM, pacemaker; PM-HFpEF, Pacemaker Heart Failure with Preserved Ejection Fraction

**Additional notes:** Exclusion criteria were selected to ensure the application of ESC HFpEF diagnostic criteria and to avoid confounding cardiac or extracardiac conditions.

Participants will undergo assessment at the screening visit, regardless of the interval since pacemaker implantation. Time since implantation will be recorded and incorporated as a covariate in descriptive analyses.

### Screening procedures and data collection

**Fig 1:**
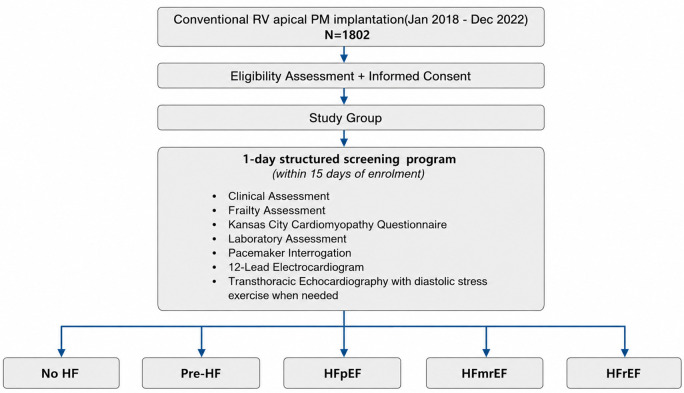
Overview of the screening program and diagnostic workflow of the PM-HFpEF study. **Abbreviations:** HF, heart failure; HFpEF, heart failure with preserved ejection fraction; HFmrEF, heart failure with mildly reduced ejection fraction; HFrEF, heart failure with reduced ejection fraction; PM, pacemaker; RV, right ventricle; KCCQ-12, Kansas City Cardiomyopathy Questionnaire–12 items. **Legend:** Patients with permanent pacemakers using right ventricular apical leads, implanted between January 2018 and December 2022, will be screened for eligibility during routine device follow-up at the study center. After exclusions and written informed consent, eligible patients will undergo a 1-day structured screening program within 15 days of enrolment. The screening protocol includes clinical assessment, frailty assessment, quality-of-life evaluation using the KCCQ-12, laboratory assessment, pacemaker interrogation, 12-lead electrocardiogram, and transthoracic echocardiography. Patients with inconclusive resting findings will undergo diastolic stress echocardiography. Patients will be classified as having no HF, pre-HF, HFpEF, HFmrEF, or HFrEF according to ESC guidelines and the Universal Definition of Heart Failure. Each participant will undergo a comprehensive assessment, which includes.

All eligible patients will be invited to participate in the screening program during a routine outpatient follow-up visit. Study procedures will be explained in detail, and written informed consent will be obtained before any study-specific assessments. All screening investigations will be completed during a one-day assessment visit, scheduled within 15 days after enrolment. An overview of the screening and diagnostic workflow is shown in **Fig 1**.

#### 1. Laboratory tests.

Non-fasting venous blood samples will be collected for laboratory parameters listed in **Table 2**.

**Table 2:** Laboratory parameters assessed for PM-HFpEF study.

Category	Parameters
**Complete blood count**	Hemoglobin, Hematocrit, RBC count, WBC count with differential, Platelet count, MCV, MCH, RDW
**Chemistry panel**	Glucose, urea, creatinine, total cholesterol, HDL, LDL, Lp(a), Triglycerides, AST, ALT, GGT, total bilirubin, serum albumin, uric acid
**Electrolytes**	Sodium, potassium, chloride, calcium, magnesium, phosphate
**Cardiac biomarkers**	NT-proBNP, hs-cTn
**Thyroid function markers**	TSH, Free T4
**Glycemic control**	HbA1c
**Inflammatory marker**	hs-CRP

**Abbreviations:** ALT, alanine aminotransferase; AST, aspartate aminotransferase; Free T4, free thyroxine; GGT, gamma-glutamyl transferase; HbA1c, glycated hemoglobin; HDL, high-density lipoprotein; hs-cTn, high-sensitivity cardiac troponin; hs-CRP, high-sensitivity C-reactive protein; LDL, low-density lipoprotein; Lp(a), lipoprotein(a); MCH, mean corpuscular hemoglobin; MCV, mean corpuscular volume; NT-proBNP, N-terminal pro–B-type natriuretic peptide; RBC, red blood cell; RDW, red cell distribution width; TSH, thyroid-stimulating hormone; WBC, white blood cell.

#### 2. Biobanking procedures.

Additional blood samples will be collected at enrollment. Samples will be processed within 60 minutes, aliquoted, labeled with a pseudonymized study identification code, and stored at −80°C with no more than one freeze–thaw cycle. Long-term storage will be performed at the Biobank of the Faculty of Medicine at the University of Porto. Sample tracking will be recorded in a secure, access-restricted electronic database, in accordance with institutional standard operating procedures (SOPs). If consent is withdrawn, all associated samples will be destroyed and removed from inventory. The detailed biobanking workflow is presented in S1 Table.

#### 3. Exploratory biomarkers.

Exploratory biomarkers of myocardial stress, fibrosis, and low-grade inflammation—soluble suppression of tumorigenicity 2 (sST2), galectin-3 (Gal-3), and interleukin-6 (IL-6)—will be measured to provide additional biological context [25,26]. These biomarkers will not contribute to diagnostic adjudication. sST2 and Gal-3 will be quantified in ethylenediaminetetraacetic acid (EDTA) plasma, and IL-6 in serum, using validated enzyme-linked immunosorbent assays (ELISAs) run in duplicate. Calibration and curve fitting will follow the manufacturer’s instructions. Samples outside the reportable range will be re-assayed after appropriate dilution. Laboratory personnel will be blinded to clinical, imaging, and device-related data.

#### 4. Echocardiography.

A comprehensive transthoracic echocardiogram (TTE) will be performed in all participants using commercially available ultrasound systems (EPIQ CVx, Philips Medical Systems, Best, The Netherlands). The protocol will include two-dimensional imaging, color and spectral Doppler, speckle-tracking strain analysis, and three-dimensional echocardiography. Image acquisition and measurement will follow recommendations from the European Association of Cardiovascular Imaging (EACVI), the American Society of Echocardiography (ASE), and joint ASE/EACVI task forces [27–29]. TTEs will be acquired by experienced cardiac sonographers and interpreted by a certified cardiologist blinded to all clinical, biomarker, and device information. Inter-observer variability will be minimized through standardized acquisition protocols, predefined measurement procedures, and periodic internal consistency checks. A 10% random sample of studies will undergo repeat measurements by a second blinded reader to quantify inter-observer variability, with discrepancies resolved by joint review. Speckle-tracking strain will be assessed using the Cardiac Motion/Mechanics Quantification (CMQ) module, with manual contour refinement if required. Global longitudinal strain (GLS) of both ventricles and left atrial strain (LAS) will be assessed according to current consensus recommendations [29–31].

In patients with HF symptoms and/or signs, elevated N-terminal prohormone of B-type natriuretic peptide (NT-proBNP) levels, and preserved LVEF, in whom elevated left ventricular (LV) filling pressures cannot be excluded at rest, diastolic stress echocardiography (DSE) will be performed to further stratify diagnosis [1,32].

#### 5. Diastolic stress echocardiography protocol.

**Fig 2:**
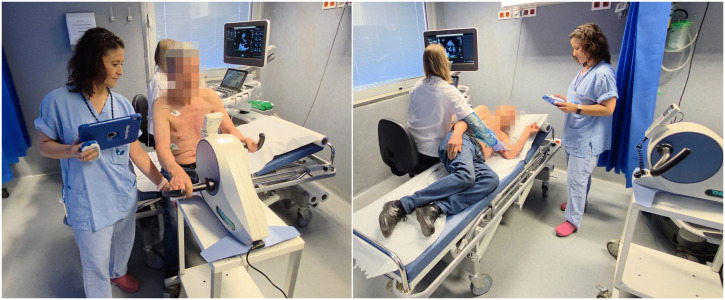
Upper-limb ergometer diastolic stress echocardiography. **Legend:** The patient is seated upright at the edge of the echocardiography bed with the upper-limb ergometer (THERA-Trainer Mobi) positioned on a removable, height-adjustable table. Once the predefined submaximal target heart rate is achieved, the table is removed to allow immediate transition to the left lateral decubitus position for apical image acquisition. The left panel illustrates the exercise phase, and the right panel the immediate post-exercise transition.

DSE will be performed using a ramped upper-body exercise protocol on an upper-limb ergometer (THERA-Trainer Mobi; Medica Medizintechnik GmbH, Hochdorf, Germany). During exercise, patients will be seated upright at the edge of the examination bed with the upper-limb ergometer positioned on a removable, height-adjustable table in front of them, as shown in **Fig 2**.

The protocol consists of short stages with predefined 30-second workload increments and standardized acquisition windows (**Table 3**).

**Table 3:** Protocol for upper-limb diastolic stress echocardiography.

Step	Description
**Adaptation phase (0)**	Level 2 workload (0.4 kg; ≈ 2 N·m), 30 s duration; patient seated upright, arms in neutral starting position.
**Incremental stages**	Up to 6 stages, with workload increasing every 30 seconds: Phase 1: Level 5 (1.0 kg, 5 N·m) Phase 2: Level 7 (1.4 kg, 7 N·m) Phase 3: Level 9 (1.8 kg, 9 N·m) Phase 4: Level 11 (2.2 kg, 11 N·m) Phase 5: Level 13 (2.6 kg, 13 N·m) Phase 6 (maximum): Level 15 (3.0 kg, 15 N·m)
**Test endpoint**	Submaximal HR target (100–110 bpm), chosen to optimize feasibility and image quality while preserving alignment with diastolic-stress frameworks [33].
**Image acquisition**	Standardized acquisition windows: • Final 30s of the highest tolerated workload (peak window) • Immediate post-exercise acquisition within 60–90 s • Apical 4- and 2-chamber, TDI (septal & lateral), TR Doppler
**Parameters measured**	Average E/e’, TR velocity, and PASP (when measurable).
**Abnormal response criteria**	E/e’ ≥ 15 and/or TRV > 3.4 m/s (or a corresponding rise in estimated PASP when measurable)

**Abbreviations:** bpm, beats per minute; E/e′, ratio of early mitral inflow velocity to mitral annular early diastolic velocity; HFA-PEFF, Heart Failure Association diagnostic algorithm for heart failure with preserved ejection fraction; kg, kilogram; N·m, Newton meter; PASP, pulmonary artery systolic pressure; s, seconds.

**Additional Notes:**

- All tests will be supervised by trained staff under continuous ECG and pacemaker intracardiac electrogram monitoring.
- Image acquisition and analysis will follow the current EACVI, ASE, and joint ASE/EACVI task force recommendations.
- Resistance levels (kg) correspond to approximate torque values (N·m); conversion to workload in Watts varies with cadence and is therefore not reported.
- Perceived exertion will be recorded at the end of the protocol using the modified Borg scale (0–10).

DSE structure is based on published diastolic-stress frameworks, including the recommendations of the European Union Seventh Framework Program (FP7)–MEDIA Initiative (EU-FP7 MEDIA), with adaptations for upper-limb exercise [33–35].

Continuous electrocardiography (ECG) and PM intracardiac electrogram monitoring will occur throughout the test. Exercise will be stopped once the predefined submaximal target heart rate of 100–110 bpm is reached, or earlier if participants experience limiting symptoms, significant arrhythmias, or request to discontinue the test [33,36]. Echocardiographic parameters will be interpreted according to the Case Definition criteria, following ESC 2021 guideline recommendations and incorporating both resting and peak-exercise thresholds [1,32]. Patients’ perceived level of exertion will be recorded at the end of the exercise using the modified Borg scale (0–10) [37].

#### 6. Clinical assessment and comorbidities.

Experienced cardiologists will perform a structured clinical assessment to assess the New York Heart Association (NYHA) functional class and document HF signs and symptoms in accordance with the Framingham criteria [1,38]. Vital signs (blood pressure, heart rate, and oxygen saturation) and cardiac auscultation will be recorded. The Heavy, Hypertensive, Atrial Fibrillation, Pulmonary Hypertension, Elder, Filling Pressure (H₂FPEF) score will be calculated [39]. Information on current medications, sociodemographic and anthropometric variables, CVRFs, and comorbidities will be collected.

Comorbidities will include coronary artery disease, prior myocardial infarction, chronic kidney disease, atrial fibrillation (AF), peripheral arterial disease, sleep apnea, pulmonary disease, thyroid dysfunction, and previous stroke. CVRFs will include hypertension, diabetes, dyslipidemia, obesity, smoking status, and alcohol consumption. The complete operational definitions are provided in S2 Table.

Health-related quality of life will be assessed using the validated Portuguese version of the 12-item Kansas City Cardiomyopathy Questionnaire (KCCQ-12) [40,41].

#### 7. Pacemaker interrogation.

Pacemaker systems from multiple manufacturers (as specified in the Study population section) will be interrogated at the screening visit using manufacturer-specific programmers.

Interrogation will provide a cross-sectional capture of device parameters, including pacing mode, atrioventricular intervals, rate-response settings (when applicable), atrial and ventricular pacing percentages, and stored atrial and ventricular arrhythmia diagnostics. Device programming will not be modified for study purposes.

For exposure analyses, pacing-related metrics and atrial tachyarrhythmia burden will be derived retrospectively from stored device diagnostics and routine device follow-up reports. A standardized 12-month observation window preceding the screening visit will be used to obtain comparable estimates of recent pacing exposure and arrhythmia burden across participants.

Given differences in detection algorithms and reporting formats across manufacturers, harmonization will focus on deriving comparable time-based exposure metrics within the predefined observation window [42,43]. When cumulative values corresponding to this window are available, they will be used directly. When only interval-based summaries are provided, window-level estimates will be derived using duration-weighted averaging across monitoring intervals to generate a single summary value per participant.

Device-detected atrial tachyarrhythmia burden (AT/AF burden) will be defined as the proportion of monitored time classified by the implanted device as atrial high-rate activity within the observation window. Episodes will be identified using manufacturer-specific diagnostics, including automatic mode-switch activation and atrial high-rate episode detection algorithms. AT/AF burden will therefore be analyzed as a device-derived measure of atrial tachyarrhythmia load rather than electrocardiographically adjudicated atrial fibrillation [42].

When available, stored electrograms will be reviewed for quality control to identify artefacts or oversensing; non-physiological detections may be flagged for sensitivity analyses.

Non-sustained ventricular tachycardia (NSVT) will be defined as ≥3 consecutive ventricular beats at a rate of >170 beats per minute, lasting <30 seconds and terminating spontaneously, in accordance with established arrhythmia definitions [44]

**8. 12-Lead electrocardiographic assessment**.

Standard 12-lead ECGs (25 mm/s, 10 mm/mV) will be processed using the VERITAS digital algorithm on the Mortara platform to obtain PR interval, QRS duration, and corrected QT (QTc) interval. Automated measurements will be used only for quality assurance. Two experienced cardiac physiologists, blinded to the automated outputs and each other’s readings, will independently measure all intervals using electronic calipers on the ByMeWeb® platform (ByMe, Porto, Portugal). Disagreements will be resolved through a joint review or adjudication by a senior reader. QTc will be corrected using Fridericia as the primary method and Bazett for predefined sensitivity analyses [45–47].

#### 9. Frailty assessment.

Frailty will be assessed using the Portuguese-validated version of the Edmonton Frail Scale, a multidimensional tool evaluating physical, cognitive, and psychosocial vulnerability [48,49]. The Edmonton Frail Scale comprises nine domains: functional independence, general health status, cognition, social support, medication use, nutrition, mood, continence, and physical performance [48]. Frailty categories will follow established scoring thresholds: fit (0–4), vulnerable (5–6), mild frailty (7–8), moderate frailty (9–10), and severe frailty (≥11) [48,49].

### Case definition and diagnostic workflow

**Fig 3:**
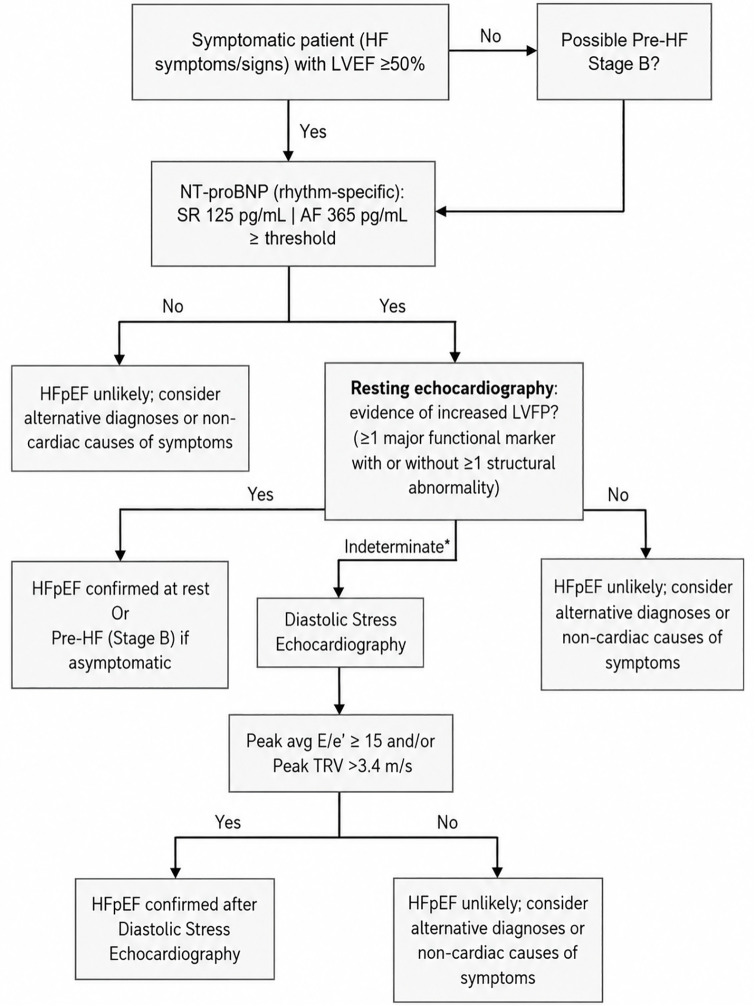
Diagnostic adjudication process: resting adjudication, diastolic stress echocardiography referral, and post-exercise adjudication. **Legend**: The stepwise screening pathway integrates rhythm-adjusted NT-proBNP thresholds, resting echocardiography, and diastolic stress echocardiography (DSE) when resting findings are inconclusive. HFpEF will be adjudicated when symptoms or signs of heart failure are present, natriuretic peptides are elevated, and at least one major functional marker of increased left-ventricular filling pressures is identified. Indeterminate cases (see S3 and S4 Tables) are referred for DSE. Abnormal DSE findings (peak E/e’ ≥ 15 and/or TRV > 3.4 m/s, or a corresponding increase in estimated PASP when measurable) are integrated with resting findings for final diagnostic adjudication. **Abbreviations:** AF, atrial fibrillation; DSE, diastolic stress echocardiography; HFpEF, heart failure with preserved ejection fraction; LAVI, left atrial volume index; LVEF, left ventricular ejection fraction; LVFP, left-ventricular filling pressures; NT-proBNP, N-terminal pro-B-type natriuretic peptide; PASP, pulmonary artery systolic pressure; TRV, tricuspid regurgitation velocity.

The diagnostic framework for HFpEF will follow the 2021 ESC Guidelines, applied stepwise, integrating resting assessment and, when required, DSE [1]. The overall diagnostic workflow is illustrated in **Fig 3**.

* *Indeterminate follows the borderline/inconclusive criteria in* S3 Table *and the adjudication pathway in* S4 Table,

#### 1. Definition of HFpEF at rest.

HFpEF at rest will be adjudicated when all the following criteria are present:

- HF symptoms and/or signs (e.g., dyspnea, orthopnea, fatigue, peripheral edema);
- LVEF ≥50%;
- Elevated natriuretic peptide levels: NT-proBNP ≥ 125 pg/mL in sinus rhythm; ≥ 365 pg/mL in AF
- Objective evidence of elevated LV filling pressures (LVFP), requiring at least one major functional marker, namely: average E/e’ > 14 or tricuspid regurgitation velocity (TRV) >2.8 m/s or pulmonary artery systolic pressure (PASP) >35 mmHg. Structural abnormalities, including left atrium (LA) enlargement (left atrial volume index (LAVI) >34 mL/m² in sinus rhythm; > 40 mL/m² in AF) and concentric remodeling or LV hypertrophy (relative wall thickness (RWT) >0.42 and/or LV mass index >95/115 g/m² [women/men]), will be recorded as supportive phenotypic markers but will not establish a diagnosis of HFpEF on their own.

#### 2. Indeterminate diagnosis at rest and referral to DSE.

Participants with LVEF ≥50%, elevated natriuretic peptide levels, and HF symptoms/signs will be referred for DSE when the resting assessment is indeterminate/inconclusive [1,32]. Resting assessment will be considered indeterminate when findings are borderline or discordant (predefined scenarios provided in S3 Table).

The Heart Failure Association Pre-test assessment, Echocardiography, natriuretic Peptides, Functional testing, and Final etiology (HFA-PEFF) score will be calculated to support diagnostic reasoning [32]. Participants with an intermediate HFA-PEFF score (2–4) will be considered eligible for DSE; however, the score will be used to inform pre-test probability and support the diagnostic pathway, and not to determine DSE referral or adjudicate HFpEF independently.

Participants who already meet the full ESC definition of HFpEF at rest will not undergo DSE. Detailed justification of all diagnostic pathways is presented in S4 Table.

#### 3. Final adjudication after DSE.

An abnormal exercise response will be defined as:

- peak average E/e’ (septal + lateral) ≥15 and/or
- peak TRV > 3.4 m/s (or a corresponding rise in estimated PASP when measurable) [32,36].

These findings will be integrated with resting parameters for the final HFpEF adjudication.

#### 4. Pre-HF (Stage B) classification.

Participants with structural abnormalities and elevated natriuretic peptides but without HF symptoms/signs will be classified as Pre-HF (Stage B) according to the Universal Definition and the American College of Cardiology/American Heart Association/Heart Failure Society of America (ACC/AHA/HFSA) 2022 staging [50].

### Ethics statement

The study will be conducted in accordance with the Declaration of Helsinki and applicable national and institutional regulations. The protocol was approved by the Ethics Committee of the Local Health Unit of Gaia and Espinho (approval reference: I22761-202306; June 2023).

Written informed consent will be obtained from all participants prior to enrollment and before any study-specific procedures. Participation will be voluntary, and individuals may withdraw at any time without affecting their clinical care.

All study data will be pseudonymized and stored in secure institutional databases with access restricted to the study investigators. Handling of personal data will comply with the General Data Protection Regulation (GDPR) and institutional governance policies.

Clinically relevant findings identified during the screening visit will be communicated to the participant’s referring physician or primary care provider to ensure appropriate clinical follow-up.

### Data collection and management

Data will be collected prospectively using standardized, study-specific assessment sheets. All variables collected during the screening visit will be entered into a secure, pseudonymized study database. Entries will follow a predefined coding structure to ensure consistency and allow reproducible export for statistical analyses. Study supervision will be carried out by the principal investigators, with routine internal checks to verify accuracy and completeness. Before analysis, the final dataset will be cleaned and formally locked.

### Statistical analysis plan

Statistical analyses will be performed using current stable versions of R and Python available at the time of analysis. Analyses will be primarily descriptive, with results reported as effect sizes and 95% confidence intervals (CIs). Where inferential tests are used to aid interpretation, they will be two-sided, and p-values will be reported alongside effect sizes and CIs and interpreted as exploratory rather than confirmatory.

The primary endpoint will be the prevalence of HFpEF among PM patients, calculated as the proportion of screened participants meeting the 2021 ESC diagnostic criteria, with exact binomial 95% CIs.

Clinical, functional, echocardiographic, and pacing-related characteristics will be compared across HF categories. Continuous variables will be summarized as mean ± standard deviation or median (interquartile range), and categorical variables as counts and percentages. Comparisons will be interpreted descriptively; formal statistical tests (ANOVA, Kruskal–Wallis, or chi-square, as appropriate) may be used to aid interpretation but will not be used to infer causal relationships.

Time since implantation will be summarized descriptively and, in supplementary analyses, key outputs may be presented across predefined clinically meaningful strata (e.g., 2–3, 3–5, and >5 years), provided subgroup sizes are sufficient for meaningful interpretation.

Associations between pacing-related parameters and HF categories (including HFpEF status) will be explored using regression analyses appropriate to the outcome scale (e.g., logistic regression for binary outcomes). These analyses will be prespecified as exploratory and hypothesis-generating and interpreted as associations rather than causal effects in this cross-sectional design. Where adjusted analyses are performed, covariates will be selected a priori based on clinical relevance and data availability; no data-driven variable selection procedures will be used.

Model diagnostics will be evaluated using standard graphical and statistical approaches. When assumptions are not met, appropriate transformations or robust alternatives will be considered. Sensitivity analyses may also explore the influence of device manufacturers and data derivation methods on device-derived metrics.

All proportions, including the proportion of participants whose clinical management is modified after screening, will be reported with 95% CIs. Sensitivity analyses will be used to assess the robustness of the HFpEF classification, with particular focus on borderline diagnostic cases.

Missing data are expected to be minimal in this study. Primary analyses will follow a complete-case approach. If missingness in key variables is non-negligible, appropriate imputation methods may be considered.

Exploratory analyses will assess associations among novel circulating biomarkers, HF categories, echocardiographic parameters, and the final adjudication of HFpEF. These analyses will be interpreted cautiously and will not undergo formal multiple-comparison adjustment.

Potential outliers will be examined using graphical methods and influence diagnostics; sensitivity analyses will be performed with and without influential observations.

A detailed Statistical Analysis Plan is provided in S5 Table.

### Trial registration

Not applicable. This observational screening study does not involve allocation to interventions.

### Status and timeline of the study

This study is ongoing, and no results have been generated. Participant recruitment began in May 2025 and is expected to be completed by June–July 2026. Data collection will continue in parallel with recruitment and is expected to conclude shortly thereafter, with final dataset completion approximately 2–3 months after recruitment closes. Statistical analyses will begin once the analytic dataset is locked, with results expected to be available by October–December 2026. All study results (aggregate analyses) will be made publicly available upon study completion, in accordance with ethical and legal requirements.

## Discussion

### Relevance of HFpEF diagnosis in pacemaker patients

Most research in PM patients has focused on pacing-induced systolic dysfunction and the development of HF with reduced ejection fraction (HFrEF) as a result of pacing-induced cardiomyopathy [51–53]. In contrast, HFpEF remains under-recognized and poorly characterized in this population, despite observational evidence suggesting that PM patients with HF may experience worse clinical outcomes [54,55]. HFpEF is likely to be common in this cohort due to multimorbidity and potential pacing-related hemodynamic effects [7,14,56,57]. As the number of PM implants increases as a consequence of demographic ageing, expanded implant indications, and improved survival, timely identification has become increasingly important [58–60].

Existing effective HFpEF therapies, including SGLT2 inhibitors and finerenone in selected patients, further reinforce this need [16,17,61,62]. Additionally, small studies in HFpEF and related diastolic phenotypes have explored pacing-rate modulation, AV-delay optimization, and physiologic pacing, showing promising but still preliminary mechanistic effects that remain insufficient to define a therapeutic role [63–65]. Without a structured diagnostic approach, HFpEF may remain unrecognized in PM patients, delaying access to guideline-directed therapy and opportunities for tailored pacemaker programming.[1,6,61,66]. Improved recognition requires accurate prevalence estimates and diagnostic pathways to address the specific challenges of paced rhythms [1,32].

Although precise estimates of HFpEF prevalence in PM patients are lacking, available evidence suggests a meaningful coexistence of HFpEF and device therapy [67,68]. For example, 19.6% of BIOPACE participants reported a history of HF, and 22.6% of hospitalized HFpEF patients in ADHERE had a permanent PM [67,68]. However, these findings result from retrospective analyses, heterogeneous diagnostic definitions, and the absence of systematic HFpEF evaluation in this group [6].

Such limitations highlight the need for prospective, standardized assessment, an unmet need that the PM-HFpEF study is designed to address using contemporary ESC criteria within a structured, real-world screening protocol.

### Rationale for using the ESC 2021 HF diagnostic definition

The ESC 2021 HF definition was chosen as the reference framework because it provides a multiparametric approach that integrates structural, functional, and biomarker domains.[1,31]. Its domain-based structure supports consistent use in routine clinical practice and across heterogeneous populations [1]. This is particularly relevant in PM patients whose multimorbidity and conduction-altered or paced rhythms frequently complicate the interpretation of isolated Doppler indices [1,28,56,68].

Alternative diagnostic algorithms, such as HFA-PEFF and H₂FPEF, are valuable probabilistic tools but pose practical limitations in PM populations [32,39]. Both rely heavily on Doppler indices (e.g., E/e´, TRV), which may be influenced by pacing-induced electrical and mechanical dyssynchrony [28,69,70]. Furthermore, evidence regarding their performance in PM cohorts remains scarce and limited to small, single-center cohorts, without formal validation as diagnostic standards in this context [71,72].

For these reasons, the ESC 2021 definition was adopted as the primary adjudication framework of this study. Its structured design supports consistent screening pathways while addressing key diagnostic challenges inherent to paced rhythms.

### Diagnostic challenges in paced patients

Paced rhythm introduces several diagnostic challenges. Atrioventricular dyssynchrony, fusion beats, and altered ventricular activation distort mitral inflow, often resulting in E/A fusion and reduced annular velocities [28,69]. In this context, E/e′ may overestimate filling pressures, and TRV can be unreliable, particularly when pacing contributes to tricuspid regurgitation [70,73]. These limitations compromise the accuracy of resting diastolic assessment. To address these challenges, the PM-HFpEF protocol incorporates DSE and LAS as supportive diagnostic measures when resting evaluation is inconclusive.

Upper-limb ergometry was chosen for DSE because it is often better tolerated in older, multimorbid adults—typical of PM populations—and allows rapid transition to supine imaging [33,35,74]. Although less extensively validated than lower-limb protocols, arm-ergometer DSE has been reported as feasible, safe, and capable of inducing sufficient cardiovascular stress to assess exercise-related changes in filling pressures [35,75].

LAS will also be incorporated as supplementary diagnostic information when Doppler indices are inconclusive. LAS has been shown to have an independent predictive value for elevated LV filling pressures [76,77]. Its inverse correlation with filling pressures has been confirmed in specific groups and indicates decreased LA compliance due to persistently high pressures [78]. However, LAS has recognized limitations in AF and lacks standardized thresholds [77]. For these reasons, it will serve only as supportive information rather than as an independent diagnostic criterion.

### Underrepresentation of pacemaker patients in HFpEF clinical trials

PM patients are consistently underrepresented in HFpEF therapeutic trials. Landmark trials such as CHARM-Preserved and I-PRESERVE lack reported prevalence of pacing [18,79]. In contrast, the TOPCAT trial included only a small proportion of PM patients, suggesting poorer outcomes in this subgroup [19]. More recent trials reinforced this pattern: DELIVER excluded individuals expected to require ≥40% ventricular pacing, and EMPEROR-Preserved allowed investigator-led exclusions based on device status [16,17]. These constraints may limit the applicability of trial evidence to PM patients in routine clinical practice.

By systematically identifying HFpEF in PM patients and characterizing their clinical and pacing profiles, the PM-HFpEF study may help refine inclusion criteria and improve the representation of this expanding patient population in forthcoming trials.

### Strengths and limitations

#### Strengths.

This study addresses a critical evidence gap by focusing on a high-risk population that has received limited research attention. The structured, ESC-aligned protocol integrates clinical, imaging, biomarker, and device-derived data. Standardized echocardiographic acquisition with blinded interpretation enhances internal consistency. Recruiting from a high-volume implantation center strengthens the clinical representativeness of the cohort. The dataset will also support exploratory analyses of HFpEF phenotypes and pacing-related features.

#### Limitations.

This single-center, cross-sectional design limits generalizability and precludes longitudinal assessment. Selection bias cannot be excluded entirely. Although standardized protocols are used, some inter-observer variability may occur. External validation in independent cohorts will be necessary to confirm applicability in broader clinical settings.

### Conclusions

To our knowledge, the PM-HFpEF study is the first prospective screening protocol specifically designed to assess HFpEF in PM patients. By applying the ESC 2021 diagnostic criteria in a real-world cohort, this study will provide robust estimates of HFpEF prevalence and a detailed characterization of the clinical, functional, and device-related profile of this high-risk population.

Beyond prevalence, the study establishes a structured diagnostic pathway tailored to the complexities of paced rhythms, integrating multimodal imaging, exercise echocardiography, and atrial strain to support confident adjudication of HFpEF.

By detecting previously unrecognized HFpEF in PM patients, the PM-HFpEF protocol may enable earlier clinical reassessment and help inform treatment pathways and trial designs that better reflect the specificities of this population.

## Supporting information

S1 Table Detailed biobanking workflow for blood sample collection, processing, and storage. (DOCX)

S2 Table Definitions and classification of comorbidities and cardiovascular risk factors. (DOCX)

S3 Table Pre-specified borderline or discordant resting findings prompting referral to diastolic stress echocardiography. (DOCX)

S4 Table Hierarchical decision framework for referral to diastolic stress echocardiography. (DOCX)

S5 Table Statistical analysis plan. (DOCX)
