# Supplementary material for: Screening for HFpEF in pacemaker patients: Study design and protocol of the PM-HFpEF study
Source: PLoS One. 2026 Jun 12;21(6):e0349667. doi: 10.1371/journal.pone.0349667 (PMC13262941; doi:10.1371/journal.pone.0349667)
Supplement: S1 Table — (DOCX) [file pone.0349667.s001.docx]

**Supporting Table 1. Detailed biobanking workflow for blood sample collection, processing, and storage**

*This table describes the standardized biobanking workflow complementing the laboratory procedures outlined in the main protocol. The workflow follows internationally recognized biobanking standards, including ISO 20387:2018, ISBER Best Practices (4th edition), and BBMRI-ERIC technical guidelines.*[1–3]

| **Procedure Step** | **Details** |
| --- | --- |
| **Blood sample collection** | Two EDTA tubes (≥3 mL each; total volume ≥6 mL). Tubes were kept upright and gently inverted according to the manufacturer’s instructions. |
| **Time to processing** | Samples processed within 60 minutes from venipuncture. |
| **Sample type** | Plasma (minimum of 6 aliquots prepared after centrifugation). |
| **Pre-centrifugation sedimentation** | 30-minute resting period at RT with tubes kept upright and undisturbed. |
| **Centrifugation** | 2000 × g for 15 minutes at RT (swing-out rotor preferred); avoid braking to prevent buffy coat disturbance. |
| **Aliquot preparation** | Six aliquots of 250 μL each, prepared immediately after centrifugation using low-retention tips. |
| **Labelling** | Each aliquot labeled with a study-specific pseudonymized ID, date and time of processing, and operator initials. |
| **Cryopreservation** | Aliquots stored at −80 °C in labeled polystyrene cryoboxes; box positions are recorded in the electronic inventory. |
| **Transport conditions** | Transfer to the biobank on dry ice with appropriate secondary containment, in accordance with institutional SOPs. |
| **Inventory and traceability** | Access-restricted electronic inventory recording sample location (freezer, box, position), date/time of processing, and operator. Traceability and data protection follow institutional SOPs and applicable data protection regulations. |
| **Freeze–thaw policy** | No more than one freeze–thaw cycle is permitted for any analytical procedure. |
| **Blinding** | Laboratory staff blinded to clinical, imaging, and device-related data. |
| **Biobank storage site** | Biobank of the Faculty of Medicine, University of Porto. |
| **Maximum storage duration** | Up to 10 years from collection or until consent withdrawal, whichever occurs first. |
| **Consent withdrawal** | Upon withdrawal of consent, corresponding samples are identified in the inventory, destroyed, and removed from the electronic records. |

**Abbreviations:** °C, degrees Celsius; EDTA, ethylenediaminetetraacetic acid; RT, room temperature; μL, microliter.

**References:**

1. Campbell LD, Astrin JJ, DeSouza Y, Giri J, Patel AA, Rawley-Payne M, et al. The 2018 Revision of the *ISBER Best Practices* : Summary of Changes and the Editorial Team’s Development Process. Biopreservation Biobanking. 2018;16: 3–6. doi:10.1089/bio.2018.0001

2. ISO 20387:2018(en), Biotechnology — Biobanking — General requirements for biobanking. [cited 6 Dec 2025]. Available: https://www.iso.org/obp/ui/en/#iso:std:iso:20387:ed-1:v1:en

3. BBMRI-ERIC,. Technical Guidelines for Biobanking: Sample Collection, Processing, Storage and Documentation. Vienna: BBMRI-ERIC; 2021. Available: https://www.bbmri-eric.eu/services/standardisation/
