# Supplementary material for: Screening for HFpEF in pacemaker patients: Study design and protocol of the PM-HFpEF study
Source: PLoS One. 2026 Jun 12;21(6):e0349667. doi: 10.1371/journal.pone.0349667 (PMC13262941; doi:10.1371/journal.pone.0349667)
Supplement: S2 Table — (DOCX) [file pone.0349667.s002.docx]

**Supporting Table 2. Definitions and classification of comorbidities and cardiovascular risk factors**

*This table lists the operational definitions used for comorbidities and cardiovascular risk factors in the PM-HFpEF study.*

| **Variable** | **Operational Definition** |
| --- | --- |
| **Hypertension** | SBP ≥140 mmHg and/or DBP ≥90 mmHg, or current use of antihypertensive medication.[1] |
| **Diabetes mellitus** | HbA1c ≥6.5%, fasting glucose ≥126 mg/dL, or use of glucose-lowering therapy.[2] |
| **Dyslipidemia** | LDL-C ≥115 mg/dL; HDL-C <40 mg/dL (men) or <46 mg/dL (women); or use of statins.[3] |
| **Obesity** | BMI ≥30 kg/m.[4] |
| **Smoking** | *Current smoker:* individuals who report smoking cigarettes at the time of assessment.  *Former smoker:* individuals who have smoked ≥100 cigarettes in their lifetime but do not currently smoke.  *Never smoker:* individuals who have smoked <100 cigarettes in their lifetime.[5] |
| **Alcohol consumption** | Consumption >14 units/week (men) or >7 units/week (women), or clinician-documented high-risk drinking (1 unit = 10 g of pure alcohol)[6] |
| **Atrial fibrillation** | *Paroxysmal:* self-terminating episodes <7 days, documented on ECG or PM.  *Persistent:* episodes ≥7 days or requiring cardioversion.  *Permanent:* continuous atrial fibrillation with no planned attempts to restore sinus rhythm.[7] |
| **Coronary artery disease / MI** | History of myocardial infarction, PCI, CABG, or ≥50% stenosis on angiography[8] |
| **Peripheral arterial disease** | ABI <0.90, imaging-confirmed arterial stenosis, or documented history of peripheral revascularization or amputation[9] |
| **Chronic kidney disease** | Clinically documented eGFR <60 mL/min/1.73m². [10] |
| **Sleep apnea** | Diagnosed obstructive or central sleep apnea (polysomnography or home test)[11] |
| **Pulmonary disease** | Chronic diagnosis of asthma, COPD, or interstitial lung disease[12] |
| **Thyroid dysfunction** | Thyroid dysfunction will be considered present if laboratory-confirmed or under treatment[13] |
| **Prior stroke / TIA** | Defined as a prior ischemic or hemorrhagic stroke or TIA documented by clinical or imaging evidence[14]. |

**Abbreviations:** ABI, ankle-brachial index; AF, atrial fibrillation; BMI, body mass index; CABG, coronary artery bypass grafting; COPD, chronic obstructive pulmonary disease; DBP, diastolic blood pressure; ECG, electrocardiogram; eGFR, estimated glomerular filtration rate; HbA1c, glycated hemoglobin A1c; HDL-C, high-density lipoprotein cholesterol; LDL-C, low-density lipoprotein cholesterol; MI, myocardial infarction; PCI, percutaneous coronary intervention; PM, pacemaker; SBP, systolic blood pressure; TIA, transient ischemic attack.

**References:**

1. Williams B, Mancia G, Spiering W, Agabiti Rosei E, Azizi M, Burnier M. 2018 ESC/ESH Guidelines for the management of arterial hypertension | European Heart Journal | Oxford Academic. Eur Heart J. 2018: 3021–3104.

2. American Diabetes Association Professional Practice Committee. 2. Diagnosis and Classification of Diabetes: Standards of Care in Diabetes-2024. Diabetes Care. 2024;47: S20–S42. doi:10.2337/dc24-S002

3. Mach F, Baigent C, Catapano AL, Koskinas KC, Casula M, Badimon L, et al. 2019 ESC/EAS Guidelines for the management of dyslipidaemias: lipid modification to reduce cardiovascular risk: The Task Force for the management of dyslipidaemias of the European Society of Cardiology (ESC) and European Atherosclerosis Society (EAS). Eur Heart J. 2020;41: 111–188. doi:10.1093/eurheartj/ehz455

4. WHO Consultation on Obesity (1999: Geneva S, Organization WH. Obesity : preventing and managing the global epidemic : report of a WHO consultation. World Health Organization; 2000. Available: https://iris.who.int/handle/10665/42330

5. Jamal A, King BA, Neff LJ, Whitmill J, Babb SD, Graffunder CM. Current Cigarette Smoking Among Adults — United States, 2005–2015. MMWR Morb Mortal Wkly Rep. 2016;65: 1205–1211. doi:10.15585/mmwr.mm6544a2

6. Wood AM, Kaptoge S, Butterworth AS, Willeit P, Warnakula S, Bolton T, et al. Risk thresholds for alcohol consumption: combined analysis of individual-participant data for 599 912 current drinkers in 83 prospective studies. Lancet Lond Engl. 2018;391: 1513–1523. doi:10.1016/S0140-6736(18)30134-X

7. Hindricks G, Potpara T, Dagres N, Arbelo E, Bax J, Blomström-Lundqvist C. 2020 ESC Guidelines for the diagnosis and management of atrial fibrillation developed in collaboration with the European Association for Cardio-Thoracic Surgery (EACTS) | European Heart Journal | Oxford Academic. Eur Heart J. 42nd ed. 2021: 373–498.

8. Knuuti J, Knuuti W, Saraste A, Capodanno D, Barbato E, Funck-Brentano C, et al. 2019 ESC Guidelines for the diagnosis and management of chronic coronary syndromes | European Heart Journal | Oxford Academic. Eur Heart J. 2020;41: 407–477.

9. Aboyans V, Ricco J, Bartelink M-E, Björck M, Brodmann M, Cohnert T, et al. 2017 ESC Guidelines on the Diagnosis and Treatment of Peripheral Arterial Diseases, in collaboration with the European Society for Vascular Surgery (ESVS) | European Heart Journal | Oxford Academic. Eur Heart J. 2018;39: 763–816.

10. Cheung AK, Chang TI, Cushman WC, Furth SL, Hou FF, Ix JH, et al. KDIGO 2021 Clinical Practice Guideline for the Management of Blood Pressure in Chronic Kidney Disease. Kidney Int. 2021;99: S1–S87. doi:10.1016/j.kint.2020.11.003

11. Kapur VK, Auckley DH, Chowdhuri S, Kuhlmann DC, Mehra R, Ramar K, et al. Clinical Practice Guideline for Diagnostic Testing for Adult Obstructive Sleep Apnea: An American Academy of Sleep Medicine Clinical Practice Guideline. J Clin Sleep Med JCSM Off Publ Am Acad Sleep Med. 2017;13: 479–504. doi:10.5664/jcsm.6506

12. Agustí A, Celli BR, Criner GJ, Halpin D, Anzueto A, Barnes P, et al. Global Initiative for Chronic Obstructive Lung Disease 2023 Report: GOLD Executive Summary. Eur Respir J. 2023;61: 2300239. doi:10.1183/13993003.00239-2023

13. Khan R, Sikanderkhel S, Gui J, Adeniyi A-R, O’Dell K, Erickson M, et al. Thyroid and Cardiovascular Disease: A Focused Review on the Impact of Hyperthyroidism in Heart Failure. Cardiol Res. 2020;11: 68–75. doi:10.14740/cr1034

14. Sacco R, Kasner S, Broderick J, Caplan L, Connors J, Culebras A, et al. An Updated Definition of Stroke for the 21st Century | Stroke. Stroke. 2013;44: 2064–2089.
