# Supplementary material for: Screening for HFpEF in pacemaker patients: Study design and protocol of the PM-HFpEF study
Source: PLoS One. 2026 Jun 12;21(6):e0349667. doi: 10.1371/journal.pone.0349667 (PMC13262941; doi:10.1371/journal.pone.0349667)
Supplement: S3 Table — (DOCX) [file pone.0349667.s003.docx]

**Supporting Table 3. Pre-specified borderline or discordant resting findings prompting referral to diastolic stress echocardiography**

*This table summarizes the pre-specified borderline and discordant resting findings that qualify as indeterminate resting assessments and prompt referral to diastolic stress echocardiography in the main protocol.*

| **Category** | **Scenario (pre-specified)** | **Rationale** |
| --- | --- | --- |
| **Functional borderline indices** | Average E/e′ of 9–14 with normal supportive structural and hemodynamic indices (normal LAVI, LV wall thickness, and TRV). | E/e′ values between 9 and 14 fall within an intermediate range in which resting filling pressures may be normal or only mildly elevated. This pattern is not sufficient to confirm or exclude HFpEF at rest, and DSE is used to determine whether filling pressures rise abnormally with exercise.[1,2] |
|  | E/e′ >14 with borderline e′ velocities (septal 6–7 cm/s, lateral 8–9 cm/s) and no structural abnormalities. | Borderline reductions in e′ velocities may occur with aging, conduction delay, and mild relaxation abnormalities and may not reliably reflect chronically elevated filling pressures in isolation. When E/e′ is elevated but supporting structural indices are normal, the overall pattern remains inconclusive, and DSE is used to assess the dynamic filling-pressure response.[1–3] |
|  | Septal–lateral e′ discordance consistent with pacing-related conduction distortion (septal e′ <5 cm/s and lateral e′ ≥8 cm/s, with Δ≥3 cm/s or septal-to-lateral ratio ≤0.6). | RV pacing and LBBB can disproportionately reduce septal e′ compared with lateral e′, making septal E/e′ less reliable for estimating filling pressures. In this setting, lateral e′ is preferred; if lateral-based E/e′ falls within a borderline range, DSE is indicated to clarify whether exercise unmasks abnormal hemodynamic responses.[1,2] |
| **Structure–function discordance** | Mild LA enlargement (LAVI 35–40 mL/m² in sinus rhythm; 40–45 mL/m² in AF) with normal E/e′ and TRV. | Mild increases in LAVI may occur from aging, prior transient pressure elevation, hypertension, or atrial arrhythmias and are not diagnostic for HFpEF in isolation. When functional indices remain non-diagnostic, DSE is used to determine whether exercise induces abnormal filling-pressure responses.[1–3] |
|  | Concentric remodeling or mild LV hypertrophy (RWT >0.42 and/or LV mass index >115 g/m² in men / >95 g/m² in women) with normal filling indices. | Concentric remodeling and mild LVH are markers of long-standing pressure load and may precede overt diastolic dysfunction. In the presence of normal E/e′ and TRV, resting findings remain inconclusive, and DSE is recommended to evaluate whether abnormal elevations in filling pressures manifest during exercise.[1,2] |
|  | TRV 2.6–2.8 m/s with otherwise non-diagnostic filling indices. | TRV values just below the ESC threshold for elevated pulmonary pressures (TRV >2.8 m/s) may underestimate true systolic pulmonary pressure because of technical or alignment limitations. When other indices are inconclusive, DSE helps assess whether pulmonary pressures rise abnormally during exercise.[1,2] |
| **NT-proBNP–echo discordance** | Rhythm-adjusted NT-proBNP ≥125 pg/mL (sinus rhythm) or ≥365 pg/mL (AF) despite non-diagnostic TTE. | Elevated natriuretic peptides suggest increased cardiac wall stress, but isolated biomarker elevation is not diagnostic of HFpEF when resting echocardiography is inconclusive. DSE is used to evaluate whether exercise unmasks abnormal increases in filling pressures consistent with HFpEF.[2,3] |

**Abbreviations:** AF, atrial fibrillation; DSE, diastolic stress echocardiography; E/e′, ratio of early mitral inflow to early diastolic annular velocity; ESC, European Society of Cardiology; e′, early diastolic mitral annular velocity; HF, heart failure; HFpEF, heart failure with preserved ejection fraction; LA, left atrium; LAVI, left atrial volume index; LVH, left ventricular hypertrophy; NT-proBNP, N-terminal pro–B-type natriuretic peptide; SR, sinus rhythm; TRV, tricuspid regurgitant velocity; TTE, transthoracic echocardiography.

**References:**

1. Nagueh SF, Smiseth OA, Appleton CP, Byrd BF, Dokainish H, Edvardsen T, et al. Recommendations for the Evaluation of Left Ventricular Diastolic Function by Echocardiography: An Update from the American Society of Echocardiography and the European Association of Cardiovascular Imaging. J Am Soc Echocardiogr. 2016;29: 277–314. doi:10.1016/j.echo.2016.01.011

2. McDonagh TA, Metra M, Adamo M, Gardner RS, Baumbach A, Böhm M, et al. 2021 ESC Guidelines for the diagnosis and treatment of acute and chronic heart failure: Developed by the Task Force for the diagnosis and treatment of acute and chronic heart failure of the European Society of Cardiology (ESC) With the special contribution of the Heart Failure Association (HFA) of the ESC. Eur Heart J. 2021;42: 3599–3726. doi:10.1093/eurheartj/ehab368

3. Pieske B, Tschöpe C, De Boer RA, Fraser AG, Anker SD, Donal E, et al. How to diagnose heart failure with preserved ejection fraction: the HFA–PEFF diagnostic algorithm: a consensus recommendation from the Heart Failure Association (HFA) of the European Society of Cardiology (ESC). Eur Heart J. 2019;40: 3297–3317. doi:10.1093/eurheartj/ehz641
