# Supplementary material for: Screening for HFpEF in pacemaker patients: Study design and protocol of the PM-HFpEF study
Source: PLoS One. 2026 Jun 12;21(6):e0349667. doi: 10.1371/journal.pone.0349667 (PMC13262941; doi:10.1371/journal.pone.0349667)
Supplement: S4 Table — (DOCX) [file pone.0349667.s004.docx]

**Supporting Table 4. Hierarchical decision framework for referral to diastolic stress echocardiography**

*This table summarizes the hierarchical decision process used to determine referral for diastolic stress echocardiography in the PM-HFpEF Study, integrating resting echocardiography, natriuretic peptides, and pre-test probability according to current ESC recommendations.*

| **Level** | **Pre-specified Scenario** | **Action** |
| --- | --- | --- |
| **Clear HFpEF (ESC 2021) at rest** | Signs/Symptoms of HF + elevated natriuretic peptides + ≥1 functional abnormality (average E/e′ >14 or TRV >2.8 m/s). | Resting findings satisfy ESC HFpEF diagnostic criteria; DSE does not modify diagnostic classification. |
| **Clear non-HFpEF** | Normal resting TTE (no major structural or functional abnormalities), natriuretic peptides within rhythm-adjusted normal limits, and absence of HF symptoms or signs. | No evidence of HFpEF or preload-related abnormalities at rest; exercise testing is not indicated. |
| **Functional borderline** | Presence of ≥1 functional borderline findings (see Supplementary Table S3):   - Average E/e′ 9–14 - TRV 2.6–2.8 m/s and E/e’≤14 - Pacing-related septal–lateral discordance with lateral E/e′ 9–14 - Borderline e′ velocities (septal 6–7 cm/s, lateral 8–9 cm/s)   With:   - Normal supportive structural and hemodynamic indices (normal LAVI, LVH, and TRV) | Exercise assessment is required to determine whether filling pressures rise abnormally during physiological stress. |
| **Discordant structural/biomarker pattern** | Any structural abnormality with non-diagnostic functional indices, or natriuretic peptide–echo mismatch, including:   - Mild LA enlargement (LAVI 35–40 mL/m² SR; 40–45 mL/m² AF) with normal E/e′ and TRV - Concentric remodeling or mild LVH with normal filling indices - Rhythm-adjusted NT-proBNP elevation with otherwise normal echocardiography | DSE is used to clarify whether exercise unmasks elevated filling pressures in these discordant cases. |

**Abbreviations:** E/e′, ratio of early mitral inflow to early diastolic annular velocity; LAVI, left atrial volume index; TRV, tricuspid regurgitant velocity; LVH, left ventricular hypertrophy; DSE, diastolic stress echocardiography; NT-proBNP, N-terminal pro-B-type natriuretic peptide; HFA-PEFF, Heart Failure Association pre-test probability score; HFpEF, heart failure with preserved ejection fraction; SR, sinus rhythm; AF, atrial fibrillation; RV, right ventricle.

**Note:** The Heart Failure Association Pre-test Probability Score (HFA-PEFF) is calculated for all participants to provide standardized pre-test probability assessment. Although informative, the score does not determine referral to diastolic stress echocardiography. Referral decisions follow the predefined indeterminate scenarios outlined in Supplementary Table S3.
