# Supplementary material for: Screening for HFpEF in pacemaker patients: Study design and protocol of the PM-HFpEF study
Source: PLoS One. 2026 Jun 12;21(6):e0349667. doi: 10.1371/journal.pone.0349667 (PMC13262941; doi:10.1371/journal.pone.0349667)
Supplement: S5 Table — (DOCX) [file pone.0349667.s005.docx]

**Supporting Table 5. Statistical Analysis Plan**

*This table details all prespecified statistical procedures used in the PM-HFpEF Study, aligned with the Statistical Analysis section of the main manuscript.*

| **Component** | **Methods and Procedures** |
| --- | --- |
| **Statistical software** | Analyses will be performed using current stable versions of R and Python at the time of analysis. |
| **Inferential framework** | Where inferential tests are presented, p-values will be reported alongside effect sizes and 95% confidence intervals (CIs) and interpreted as exploratory rather than confirmatory. No prespecified claims of statistical significance will be made. |
| **Primary endpoint** | Prevalence of HFpEF, calculated as the proportion of screened participants fulfilling ESC 2021 diagnostic criteria. Exact binomial 95% CIs will be provided. |
| **Descriptive statistics** | Continuous variables: mean ± SD or median (IQR), according to distribution.  Categorical variables: counts and percentages. |
| **Group comparisons** | Differences across HF categories (No HF, Pre-HF, HFpEF, HFmrEF, HFrEF) may be explored using ANOVA or Kruskal–Wallis tests for continuous variables and χ² tests for categorical variables. Results will be interpreted descriptively, and post-hoc comparisons, when presented, will be interpreted without formal multiplicity correction. |
| **Associative analyses** | Associations between pacing-related parameters and HF categories will be explored using regression models appropriate to the outcome scale and distribution (e.g., logistic, ordinal, or other generalized linear models).  Covariates in adjusted models will be selected a priori based on clinical relevance and data availability.  Model assumptions will be evaluated using graphical and statistical diagnostics; transformations or robust alternatives will be considered when required.  These analyses will be interpreted as associative and hypothesis-generating rather than predictive or causal. |
| **Exploratory analyses** | Exploratory analyses will assess associations between novel circulating biomarkers, HF categories, echocardiographic measures, and final adjudication of HFpEF.  These analyses will be interpreted cautiously and will not undergo formal adjustment for multiple comparisons. |
| **Proportions of interest** | All proportions—including the proportion of participants whose clinical management is modified following screening—will be reported with 95% CIs. |
| **Sensitivity analyses** | Sensitivity analyses will assess the robustness of HFpEF classification, particularly in borderline diagnostic cases and when excluding participants with suboptimal data quality. Additional sensitivity analyses may explore the influence of device manufacturer and data derivation method on device-derived metrics. |
| **Handling of missing data** | Missing data are expected to be minimal given the standardized single-visit screening protocol.  Primary analyses will follow a complete-case approach.  The extent of missingness will be reported descriptively. If missingness in key variables is non-negligible, appropriate imputation methods may be considered in sensitivity analyses. |
| **Outlier handling** | Potential outliers will be examined using graphical methods and influence diagnostics. Observations will only be excluded if identified as clear data-entry errors. Skewed variables (e.g., NT-proBNP) may be log-transformed where appropriate. |
| **Reporting standards** | Analyses and reporting will follow STROBE and PLOS ONE guidelines. |

**Abbreviations:** AF, atrial fibrillation; CI, confidence interval; DSE, diastolic stress echocardiography; ESC, European Society of Cardiology; HF, heart failure; HFmrEF, heart failure with mildly reduced ejection fraction; HFpEF, heart failure with preserved ejection fraction; HFrEF, heart failure with reduced ejection fraction; IQR, interquartile range; NT-proBNP, N-terminal pro-B-type natriuretic peptide; SD, standard deviation.
